# Design and Evaluation of LYSO/SiPM LIGHTENING PET Detector with DTI Sampling Method

**DOI:** 10.3390/s20205820

**Published:** 2020-10-15

**Authors:** Zhenzhou Deng, Yushan Deng, Guandong Chen

**Affiliations:** School of Information Engineering, Nanchang University, Nanchang 330031, China; 401030718028@email.ncu.edu.cn (Y.D.); 411014418231@email.ncu.edu.cn (G.C.)

**Keywords:** LIGHTENING PET detector, silicon photomultiplier, readout circuit, dual time interval, light guide design, flood histogram, energy resolution, coincidence timing resolution

## Abstract

Positron emission tomography (PET) has a wide range of applications in the treatment and prevention of major diseases owing to its high sensitivity and excellent resolution. However, there is still much room for optimization in the readout circuit and fast pulse sampling to further improve the performance of the PET scanner. In this work, a LIGHTENING${}^{®}$ PET detector using a 13 × 13 lutetium-yttrium oxyorthosilicate (LYSO) crystal array read out by a 6 × 6 silicon photomultiplier (SiPM) array was developed. A novel sampling method, referred to as the dual time interval (DTI) method, is therefore proposed to realize digital acquisition of fast scintillation pulse. A semi-cut light guide was designed, which greatly improves the resolution of the edge region of the crystal array. The obtained flood histogram shown that all the 13 × 13 crystal pixels can be clearly discriminated. The optimum operating conditions for the detector were obtained by comparing the flood histogram quality under different experimental conditions. An average energy resolution (FWHM) of 14.3% and coincidence timing resolution (FWHM) of 972 ps were measured. The experimental results demonstrated that the LIGHTENING${}^{®}$ PET detector achieves extremely high resolution which is suitable for the development of a high performance time-of-flight PET scanner.

## 1. Introduction

Positron emission tomography (PET) has been pursuing better resolution and shorter imaging time due to its high application value in the early diagnosis of tumors, cardiac function assessments, nervous system analysis, etc. [1,2,3]. Specifically, a PET system with a higher resolution can achieve a more accurate lesion location and efficient rejection of the events outside the region of interest. Moreover, shorter imaging time can greatly accelerate the examination time and reduce the pain of patients [4]. The performance of a PET system [5,6,7,8,9] directly depends on its components, which include a scintillation crystal, a photodetector, a readout circuit, etc.

On the one hand, as the device that detects photons and converts photons into an electrical signal, the response speed and resolution of the photodetector directly determine the performance of the PET detector. In addition, the size of the photodetector is the main factor affecting the integration of the PET system. The position sensitive photomultiplier tube (PSPMT) was used in the early PET system as the photodetector for photoelectric conversion [10,11,12]. However, the performance uniformity of PSPMT is poor due to its working principle. The miniaturization of a PET system is seriously limited as a result of the large volume and high working voltage of PSPMT. A silicon photomultiplier (SiPM), also referred to as a multi pixel photon counter (MPPC), according to the operation principle, as a new type of semiconductor device, is gradually replacing the PSPMT in a new generation of photodetectors in PET systems [13,14,15,16,17] based on the advantages of high quantum efficiency, high gain, fast time response, low operating voltage, compact structure, and immunity to magnetic fields [18,19,20].

On the other hand, as the material that absorbs high-energy photons and converts them into visible light photons, scintillation crystals are required to have the characteristics of fast decay time, high light output, excellent energy resolution, and high density [21,22]. Lutetium-yttrium oxyorthosilicate (LYSO), as an inorganic scintillation crystal material, has stable physical and chemical properties, is not deliquescent, and has high detection efficiency for gamma rays to sufficiently meet those requirements of a PET detector [23,24,25].

Due to these prominent advantages, the SiPM array coupled with LYSO crystal system has gained an unprecedented upsurge of attention. SiPM arrays one-to-one coupled with crystals have been applied in PET detectors and obtained good time performances [26,27,28]. However, the spatial resolution of the detector is limited by the size of the crystal pixel. Even worse, an SiPM array one-to-one coupled with an LYSO crystal will need more readout channels, which will seriously increase the burden of subsequent sampling circuits and system cost. In order to reduce the cost of the SiPM array and crystals and the complexity of the readout circuit, a SiPM unit is usually coupled with multiple crystals and a multiplexing method is utilized to reduce the readout channels. At the same time, the time performance of the detector is deteriorated [29,30,31,32].

In recent years, numerous efforts have been devoted to developing high performance PETs with various SiPM arrays coupled with LYSO crystals. Specifically, Xu et al. [33] developed a time-of-flight (TOF) PET detector by multiplexing 64 fast outputs and 64 standard outputs into a few channels. Melroy et al. [34] developed a head-mounted micro-dose PET brain imager by utilizing twelve SiPM PET modules. Ascenzo et al. [35] built a proton therapy monitoring system based on PET that utilized plug and imaging (P&I) technology. Poladyan et al. [36] developed a small-scale PET system and used the truncated center of gravity (TCoG) and raised to the power (RTP) methods for reconstruction of the flood histogram. Du et al. [37] developed a proof-of-concept detector module, which contained nine detector submodules, by utilizing the shared-photodetector readout method. Han et al. [38] developed a dual-ended depth-of-interaction (DOI)-TOF PET module using a charge division circuit. Wei et al. [39] designed a detector based on a sparse SiPM array and a side-by-side phoswich crystal array which was composed of LYSO and ${\mathrm{Gd}}_{3}{\mathrm{Al}}_{2}{\mathrm{Ga}}_{3}O_{12}$ (GAGG). Kuang et al. [40] coupled two SiPM arrays at both ends of the LYSO crystal array to form a double-ended readout PET detector. Table 1 summarizes detailed specifications and performances of the detectors developed in the previous studies.

The detectors developed in the previous study have achieved excellent performances. However, the resolution and integration of the PET system can be further enhanced by designing better readout circuits. It is still a potential challenge to find a more effective pulse sampling method that can obtain pulse information accurately with reasonable cost and power consumption.

To further improve the performance and simplify the design of PET system, in this work, we developed a LIGHTENING${}^{®}$ PET detector based on a 13 × 13 LYSO array, a 6 × 6 SiPM array, and a semi-cut light guide, and evaluated the performance of the detector comprehensively. The main contributions of this paper are summarized as follows.

(1) A novel sampling method related to the dual time interval (DTI) method is presented. The method uses a narrow time interval to sample the fast leading edge by employing a few discriminators with programmable voltage thresholds to determine the time points when the scintillation pulse crosses the set thresholds. The wide time interval used to sample the tail edge of the scintillation pulse employs a conventional analog-to-digital (AD) chip. The method effectively realizes the sampling of fast scintillation pulses while significantly reducing the hardware cost.
(2) A multiplexing method was designed to multiplex the fast output by symmetric charge division (SCD) circuit and multiplex standard output via a signal driven multiplexing (SDM) circuit. The number of readout channels and the complexity of subsequent acquisition circuits are greatly reduced with the multiplex circuit.
(3) A semi-cut light guide with a gap cut in the middle of the second crystal pixel at the edge was designed, and it enhanced the resolution ability of crystal pixels on the edge of the crystal array significantly.
(4) A gain adaptive method based on adjustable resistance is proposed to minimize the gain difference of different SiPM channels and improve the uniformity of the flood histogram.

In Section 2, we first describe the basic hardware composition and circuit design of the detector. Then we introduce the data processing and transmission methods in detail. In Section 3, we describe the experiments, and evaluate the flood histogram quality, spatial resolution, energy resolution, and time resolution of the detector. The final conclusions and discussion are in Section 4.

## 2. Detector Design and Data Processing Method

### 2.1. General Description

**Figure 1:**
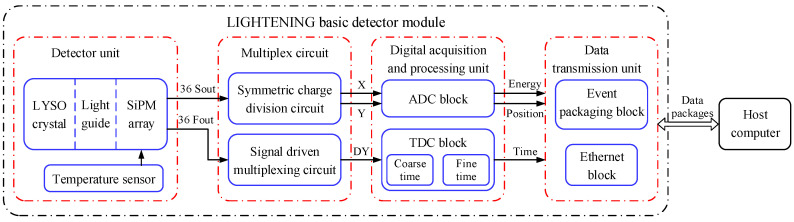
The scheme of instrumentation setup of the LIGHTENING${}^{®}$ PET basic detector module.

**Figure 2:**
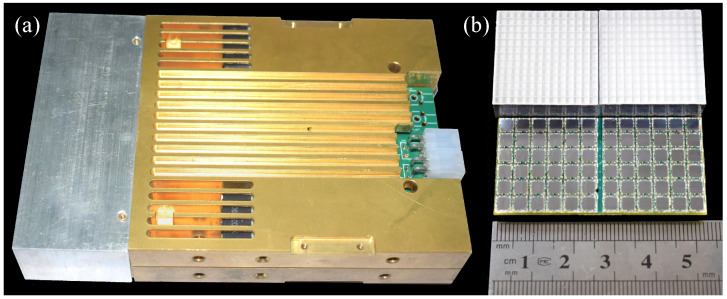
Photographs of the BDM (**a**) and 13 × 13 LYSO crystal array with the 6 × 6 SiPM array (**b**).

LIGHTENING${}^{®}$ PET basic detector module (BDM) is a detector module used for a small animal imaging scanner, as shown in Figure 1, which consists of four basic units, including the detector unit (DU), the multiplex circuit (MC), the digital acquisition and processing unit (DA&PU), and the data transmission unit (DTU). A BDM (Figure 2a) consists of a 1 × 4 array of detector units, each of which consists of a LYSO array, a semi-cut light guide, and a SiPM array, as shown in Figure 2b. The scintillation pulses generated from the detector unit are amplified and then multiplexed into a few signals by the multiplexing circuit. The digital acquisition and processing unit, which consists of a time-to-digital converter (TDC) block and an analog-to-digital converter (ADC) block, samples the signal and extracts the time, energy, and position information of the pulses. The data transmission unit packages the information and transmits it to the host computer through the Gigabit network line. Then the energy calibration and qualification, crystal identification, and coincidence detection are performed on the host computer to reconstruct the location of the annihilation event.

**Figure 3:**
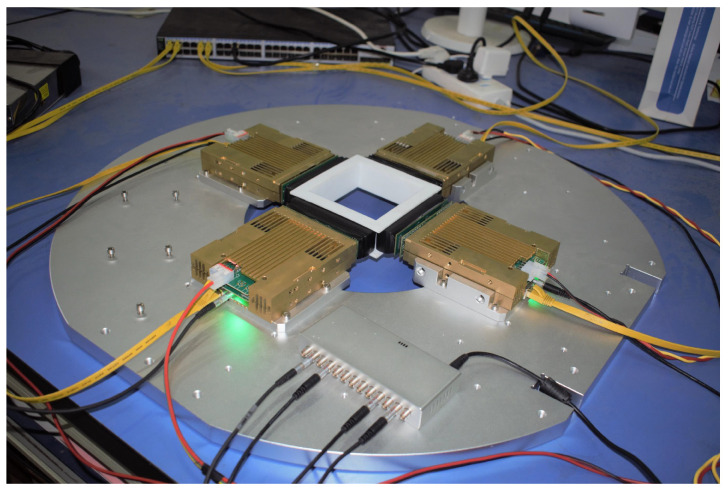
Our small-animal PET scanner employs 4 BDMs.

With the modular design, the LIGHTENING${}^{®}$ detector can be used and maintained conveniently. As shown in Figure 3, we employed 4 BDMs arranged as a quadrilateral ring to build a small-animal PET scanner. The 4 BDMs were synchronized by a high precision global clock. The general specifications of LIGHTENING${}^{®}$ PET are shown in Table 2. In this paper, we focus on describing the design and implementation of the DU, MC, DA&PU, and DTU, and reporting the performance properties measured of the LIGHTENING${}^{®}$ PET BDM.

### 2.2. The Detector Unit

The detector unit is composed of an LYSO crystal array, which is coupled to a 6 × 6 SiPM array consisting of SensL MicroFC-30035 via a semi-cut light guide block. The package dimensions of the FC30035 are 4 mm × 4 mm, and the active area is 3 mm × 3 mm. The overall surface size of the SiPM array is 25 mm × 25 mm with the minimum spacing of 0.2 mm for better collection of photons. The crystal pixels of 1.92 mm in size are selected to form a 13 × 13 crystal array under the trade-off between the spatial resolution and the system cost of the PET detector [41,42]. Every crystal pixel is polished on six sides to reduce the transmission loss of scintillation photon. The BaSO${}_{4}$ powder of 0.1 mm thickness fills the space between the adjacent crystal to reflect the scintillation light and isolate each crystal optically. The outmost layer except for the coupling surface of the crystal array is wrapped in a 0.2 mm thick aluminum foil to isolate the photons in the environment. Since SiPM is sensitive to temperature, the gain, dark current, etc., will vary with temperature change. To adjust the gain according to the current temperature, a temperature sensor is placed on the back of the SiPM board to feedback the temperature of the SiPM array in real time. The integrated digital temperature sensor LM71 was selected with an output accuracy of 0.07 ${}^{\circ }C$.

**Figure 4:**
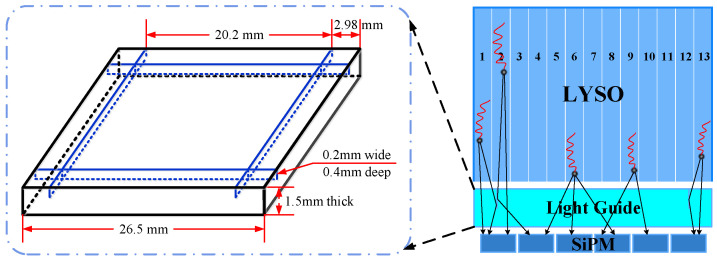
Illustration of the semi-cut light guide design and the photon transport in edge crystals with the semi-cut light guide.

Due to the surface area of the crystal array being larger than that of the SiPM array, most of the photons produced by the outermost crystal pixel could not be detected in the case that the crystal is directly coupled with SiPM. The loss of photons would cause position misjudgment, which would finally reduce the image quality. To ensure that all the photons were detected effectively, we added a light guide between the crystal and SiPM. The surface area of the light guide was 26.5 mm × 26.5 mm, the same as that of the crystal array. The thickness of the light guide was also of great significance because it would directly affect the uniformity and dynamic range of the flood histogram. In this study, a 1.5 mm thick light guide was used to make a compromise between the photon diffusion and the photon loss. The material of the light guide was optical acrylic with the higher refractive index. We added cutting gaps in the light guide to further improve the identification of crystals in the edge region [43,44,45]. The design schematic diagram of the light guide is shown in Figure 4. A gap of 0.2 mm wide and 0.4 mm deep was cut in the middle of the second crystal pixel at the edge. We used BaSO${}_{4}$ powder in the gap, which is functionally similar to the powder among the crystal pixels. The facet with the slit of the light guide was coupled with the light-exiting surface of the crystal array, and the side without a slit was coupled with the SiPM array. The four sides of the light guide were painted black to isolate photons outside. The photon transport in edge crystals with the semi-cut light guide is shown in Figure 4.

### 2.3. Multiplex Circuit

**Figure 5:**
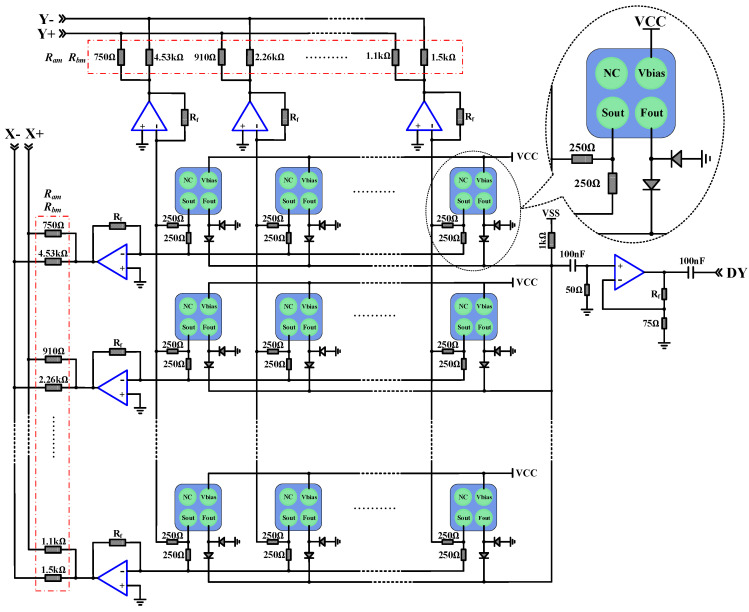
Schematic of the multiplex circuit for SiPM array F${}_{o}{}_{u}{}_{t}$ signals and S${}_{o}{}_{u}{}_{t}$ signals.

The readout board connected with the SiPM array contained a multiplex and amplified circuit. A common bias voltage was supplied to all the SiPMs. Standard output (S${}_{o}{}_{u}{}_{t}$) of SiPMs was utilized to obtain position information and energy information of gamma photons, and fast output (F${}_{o}{}_{u}{}_{t}$) was utilized to obtain time information. Since the rise time of F${}_{o}{}_{u}{}_{t}$ is much faster than that of S${}_{o}{}_{u}{}_{t}$, which could greatly improve the time performance. To further reduce the number of readout channels, as shown in Figure 5, a symmetric charge division (SCD) circuit was used to multiplex S${}_{o}{}_{u}{}_{t}$ signals, and a signal driven multiplexing (SDM) circuit was used to multiplex F${}_{o}{}_{u}{}_{t}$ signals [46,47,48]. In addition, an operational amplifier was used in the multiplex circuit to amplify the signal in certain proportions, since the output signal of a SiPM is quite weak.

The S${}_{o}{}_{u}{}_{t}$ signals of the SiPM array are divided into two components through two resistors and connected to x and y directions respectively. The x-direction signals of the same row and the y-direction signals of the same column are sent to the operational amplifier respectively. The 6 × 6 SiPM signals are converted into 6 + 6 row and column signals. The operational amplifier and its feedback resistor formed a reverse amplifier circuit to amplify the row and column signals. After a specified value of resistance, the row and column signals are converted into four corner signals with a specified magnification. To ensure that the total magnification of the row and column signal after amplification is consistent, the specified resistance values should be limited. The specified resistance value is calculated by Equations (1) and (2):

(1) $$R{}_{a}{}_{m}=\frac{R_{max}}{\left(m-1\right)\frac{E-1}{M-1}+1}\;\;,$$

(2) $$R{}_{b}{}_{m}=\frac{R_{max}}{\left(M-m\right)\frac{E-1}{M-1}+1}\;\;,$$

where *R${}_{m}{}_{a}{}_{x}$* is the maximum value in the resistor network, *M* is the number of channels, *m* is the channel number (range from 0 to M), and *E* is the ratio of the expected maximum resistance to the minimum resistance. In this design, set *R${}_{m}{}_{a}{}_{x}$* = 4.53 kΩ, *M* = 6, and *E* = 6, to get the resistance value calculated by Equations (1) and (2) as shown in Figure 5. A small package chip resistor with 1% precision was selected for this application to minimize the detector volume. The current feedback amplifier AD8012 was selected as the operational amplifier, which is low power and capable of providing 350 MHz bandwidth while using only 1.7 mA per amplifier.

As shown in Figure 5, the F${}_{o}{}_{u}{}_{t}$ signals of SiPM are isolated by two diodes to prevent signal backflow. Then all the signals are connected together by wires to form a common fast output signal. The fast output signal is amplified in phase by an operational amplifier into a DY signal. The common time information of the detector was extracted form the DY signal. The influences of different SiPM channels’ distribution parameters and dark noise can be effectively reduced by utilizing the diodes; hence, the time performance can be improved. The Schottky diode RB751SL with a fast recovery time of 8 ns was selected to reduce the transmission distortion. Its package dimensions (1.0 × 0.6 mm${}^{2}$) are extremely small and thin, which can greatly save hardware design space.

### 2.4. The Digital Acquisition and Processing Unit

The typical rise time of the DY signal is about 6 ns. An expensive analog-to-digital converter with a sampling rate of 1 to 2 Gsps is required to obtain the characteristics of the rising edge of the scintillation pulse. The cost of a single channel would be very high. In the PET system, there are a large number of channels. As a result, the total cost and the power consumption of the system would be too large to bear.

**Figure 6:**
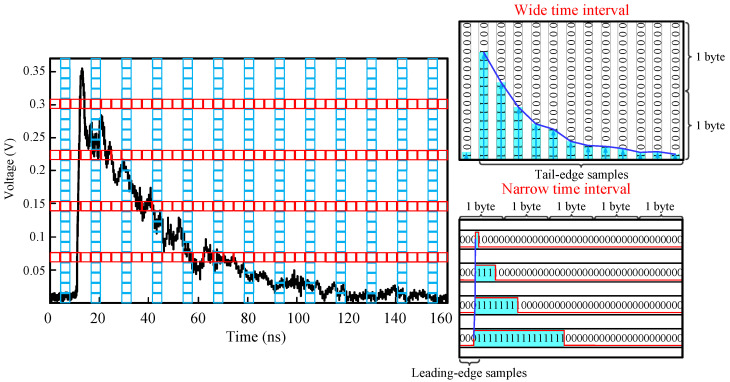
Illustration of the DTI method. The narrow time interval (horizontal) used to sample the fast leading edge by employs a few discriminators with programmable voltage thresholds to determine the time points when the scintillation pulse crosses the set thresholds. The wide time interval (vertical) used to sample the tail edge of the scintillation pulse employs a conventional analog-to-digital (AD) chip.

To reduce the high cost of the sampling circuit for the rising edge of the scintillation pulse, we propose the DTI method as an alternative scheme to the high sampling rate analog-to-digital converter. As shown in Figure 6, the DTI method used a narrow time interval in the TDC block to sample the fast leading edge and a wide time interval in the ADC block to sample the tail edge of the scintillation pulse. The DTI method employs a few discriminators with programmable voltage thresholds to determine the time points when the scintillation pulse crosses the set thresholds. The voltage-time sample replaces the conventional time-voltage sample. Therefore, the sampling of the rising edge can be realized with lower cost by using several discriminators with a programmable voltage threshold in one field programmable gate array (FPGA).

The TDC block was realized by adder carry chain composed of a logic unit inside the FPGA, and it was utilized to sample the DY signal to obtain timing information with high quality. The TDC integrated inside FPGA can maximize the use of FPGA resources, which can not only greatly reduce the cost of the system, but also increase the system’s flexibility. The acquisition of time information includes coarse time and fine time. Coarse time is obtained by counting the accurate external clock signal, namely, the value is the product of counting and clock period, which can guarantee long time measurement. The data output from the carry chain is latched when the rising edge of the clock comes, and then the latched data are decoded to obtain the fine time information, which can get the finer time accuracy.

**Figure 7:**
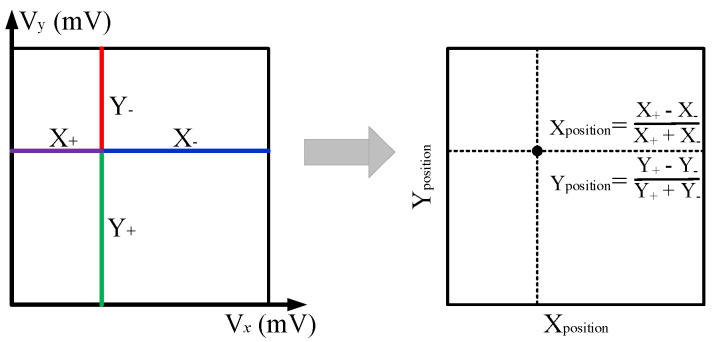
Illustration of position calculation with the Anger Logic algorithm.

The ADC block samples the x and y signals with a wide time interval. The conventional analog-to-digital (AD) was sufficient to get enough sampling points of the tail edge and reconstruct the pulses. The energy information is obtained by summing the charge of the pulse. The Anger Logic method is used to decode the position information owing to the readout channels being multiplexed. As shown in Figure 7, the position information of the scintillation pulse is calculated by Equations (3) and (4):

(3) $$X{}_{p}{}_{o}{}_{s}{}_{i}{}_{t}{}_{i}{}_{o}{}_{n}=\frac{X_{+}-X_{-}}{X_{+}+X_{-}}\;\;,$$

(4) $$Y{}_{p}{}_{o}{}_{s}{}_{i}{}_{t}{}_{i}{}_{o}{}_{n}=\frac{Y_{+}-Y_{-}}{Y_{+}+Y_{-}}\;\;.$$

**Figure 8:**
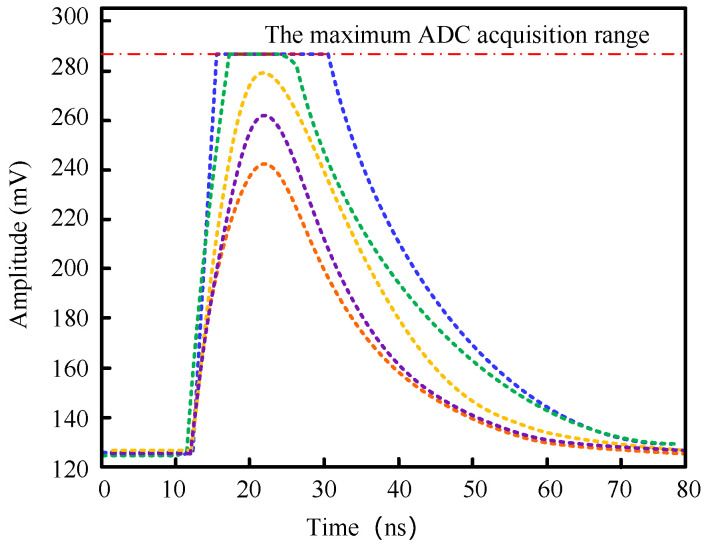
The top of the pulse is cut off when the pulse exceeds the maximum dynamic range of the ADC.

A LIGHTENING${}^{®}$ PET detector consists of four SiPM arrays that are contained 144 pieces of SiPM. The gain difference among different channels will affect the performance uniformity of the detector, and ultimately lead to the deterioration of the imaging quality. Therefore, a gain adaptive module was added to the digital acquisition and processing unit to adjust the gain of each channel and make the best use of the performance of the ADC. The basic principle of the adaptive algorithm is to achieve large amplification by utilizing the operational amplifier, and then use the resistance network formed by the programmable resistance to realize the voltage distribution. Specifically, the gain of the current channel is set to the maximum. At the same time, some scintillation pulses are collected to judge whether the current overflow ratio exceeds the maximum dynamic range of ADC. As shown in Figure 8, when the pulse exceeds the maximum dynamic range of the ADC, the top of the pulse will be cut off. The gain is reduced by adjusting the value of the digital potentiometer, if the overflow ratio exceeds the set value. After that, the data acquisition and judgment operations are repeated until the overflow ratio meets the requirements. Each channel performs the gain adaptive adjustment until the adjustment completes in the final channel, and the detector enters the normal data acquisition mode. In this design, AD8039 was selected as the operational amplifier of the adaptive module, which is a high speed (350 MHz) voltage feedback amplifier with an exceptionally low quiescent current of 1.0 mA per the typical amplifier (1.5 mA maximum). The adjustable resistor is a digital potentiometer with 256 linear adjustable ranges.

### 2.5. The Data Transmission Unit

**Figure 9:**
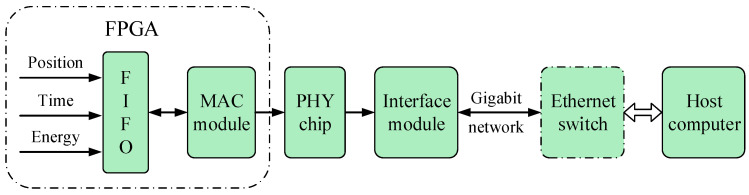
Illustration of the data transmission process.

The scintillation pulse data are packaged and compressed into a specific format after digitization and processing, which include the detector’s unit number, time information, position information, energy information, and temperature information of the SiPM array. The data packages are stored in the first input first output (FIFO) module of FPGA. The media access control (MAC) module packs the data according to user datagram protocol (UDP) and writes it into physical (PHY) chip, which is implemented by Marvell’s 88E1111 chip and its peripheral circuit. PHY chip drives the interface module and transmits the data to the host computer through the Gigabit network cable for further processing. The BDMs are paralleled on the switch and output to the host computer. The data transmission process is as shown in Figure 9. Furthermore, the data transmission unit can also respond to the specific request from the host computer, which mainly includes modifying the detector configuration information, updating the firmware program online, etc.

## 3. Experiment and Results

### 3.1. Experiment Setup

**Figure 10:**
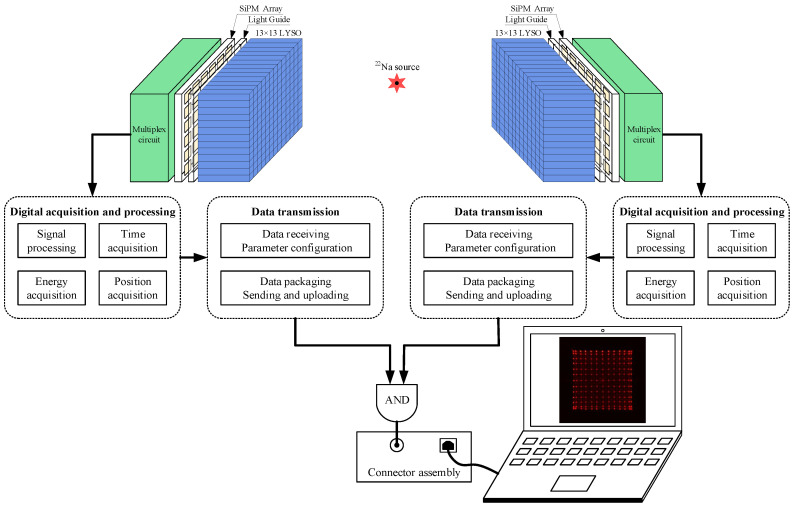
Figure illustrating the experimental setup in the coincidence mode.

To find the best working conditions of the detector and evaluate the optimal performance of the PET detector, we carried out experiments to obtain detector event data. All the experiments were carried out in a dark box with temperature constantly. Except for the experiments with temperature as a variable, all experiments were carried out at 5 ${}^{\circ }C$. A ∼20μCi ${}^{2}{}^{2}$Na was used as the radiation source. The common bias voltage of SiPM was set at 28.8 V. Firstly, we used a SiPM array to evaluate the flood histogram with the detector operating temperature as a variable. The output precision of the temperature sensor was rounded to 0.1 ${}^{\circ }C$, since only three bits of the output data frame were used to store the temperature information. The DTI method was used to sample the original pulse, and the comparison level was set as 4, 8, 12, and 16 mV. The source was located 30 mm away from the center of LYSO crystal array. Then we evaluated the flood histogram under different overflow ratios to find the ADC overflow ratio which makes the detector work at the optimal state. The experimental setup for measuring the time performance of the developed detector module is shown in Figure 10. Two BDMs were placed opposite each other and the source was placed in the center between the two detectors with a distance of 25 mm. Detector pairs work in the coincidence mode, ensuring that the maximum number of coincidence events are collected.

### 3.2. Flood Histogram and Spatial Resolution

**Figure 11:**
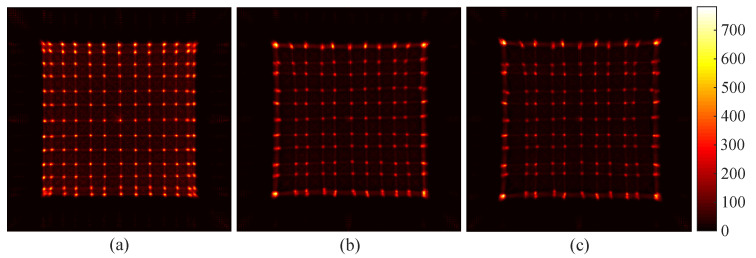
The flood histogram of a coupled semi-cutting light guide for 13 × 13 crystals and a SiPM array (**a**); a coupled continuous light guide for 13 × 13 crystals and a SiPM array (**b**); and directly coupled 13 × 13 crystals with a SiPM array (**c**).

We carried out experiments to obtain the flood histogram by utilizing different light guides to verify the availability of the light guide design. The flood histograms obtained by utilizing different designs of light guides are shown in Figure 11. To display differences of the flood histograms more intuitively, we added a color bar to use a different color to represent the count of each pixel. Figure 11a shows the flood histogram obtained by using the semi-cut light guide designed in this paper. It can be seen that both the center and the edge crystal pixels of the crystal array are clearly separated. This is the result of the design of a semi-cut gap that increases the refraction of photons. Figure 11b shows the flood histogram obtained by coupling a continuous light guide with a thickness of 1.5 mm between the crystal array and the SiPM array. The two outermost rows and columns of the flood histogram are about to separate but not completely separated. Figure 11c shows the flood histogram obtained by coupling the crystal array directly to the SiPM array. Only the central crystal pixels are distinguishable, and crystal pixels in the outermost two rows and columns are still stuck together. Moreover, the uniformity of the flood histogram without the light guide was worse than that of the flood histogram obtained with the continuous light guide. The optimal flood histogram uniformity was obtained with the use of a semi-cut light guide in terms of both the brightness of the spots and the distances among the spots.

**Figure 12:**
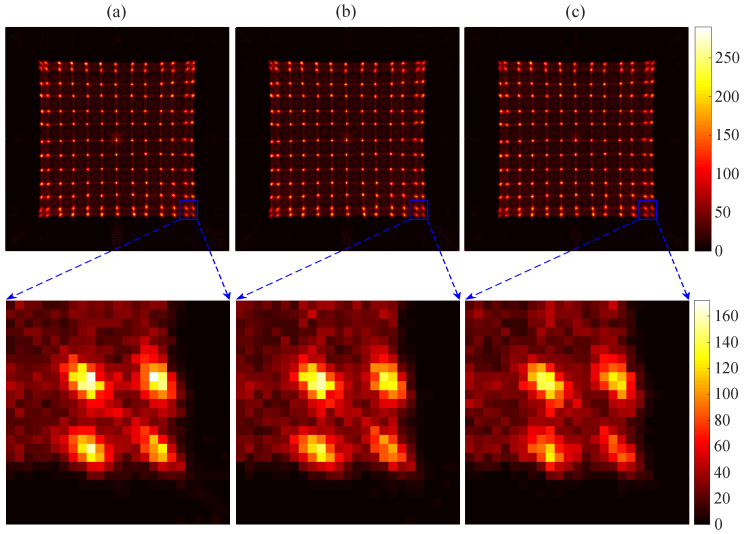
The flood histograms obtained at temperatures of 5 ${}^{\circ }C$ (**a**), 15 ${}^{\circ }C$ (**b**), and 25 ${}^{\circ }C$ (**c**). The four spots at the edge of the flood histogram are given to observe the differences of the spots.

**Figure 13:**
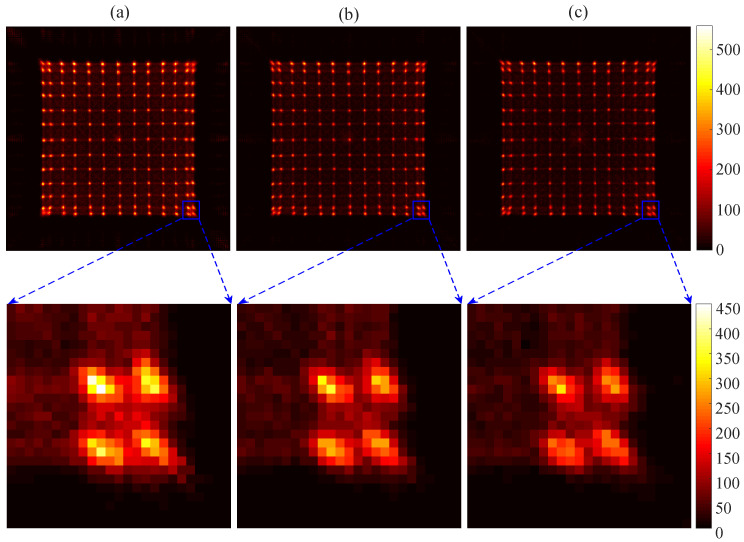
The flood histograms obtained with ADC overflow ratios of 5% (**a**), 30% (**b**), and 60% (**c**). The four spots at the edge of the flood histogram are given to observe the differences of the spots.

To study the influence of temperature and ADC overflow ratio to the flood histogram, we set up experiments to obtain the flood histogram under different temperatures and different ADC overflow ratios, respectively. Firstly, we set the operating temperature of the circuit board as a variable. Figure 12 shows the flood histogram at temperatures of 5 ${}^{\circ }C$, 15 ${}^{\circ }C$, and 25 ${}^{\circ }C$. We enlarged the four spots at the edge of the flood histogram separately to display the differences in spot brightness. Then, we changed the ADC overflow ratio to obtain the flood histogram with a different overflow ratio. Figure 13 shows the flood histogram with the overflow ratios of 5%, 30%, and 60%. Similarly, we enlarged the four spots at the edge of the flood histogram; there we can observe some slight changes of spot brightness.

**Figure 14:**
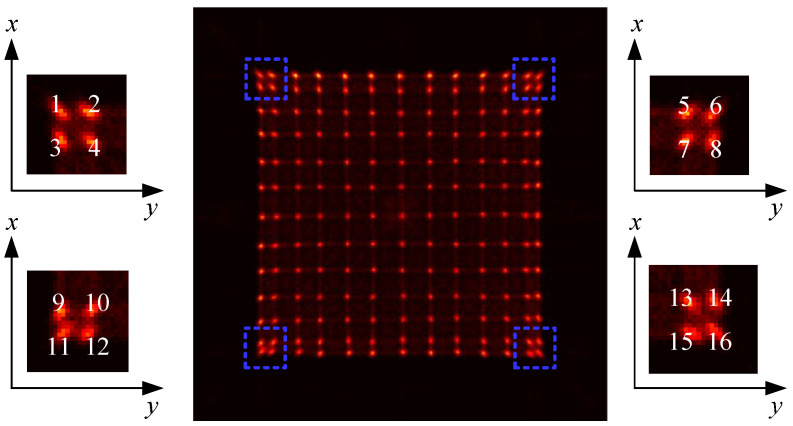
Figure illustrating how the flood histogram quality parameter was calculated.

To quantitatively evaluate the quality of flood histogram under different temperatures and overflow ratios, an evaluation method is proposed. Specifically, for the flood histogram, the spot of each crystal should be concentrated as much as possible, while the spots of two adjacent crystals should be separated as much as possible. However, it is difficult to distinguish the crystal pixels at the four corners due to the geometry of the crystal and SiPM array. With a reasonable design of the light guide, we achieved a good resolution in the edge crystal pixels. However, the resolution of the crystals on the corner is still slightly inferior to that of the central crystal. To some extent, the resolution of the crystals on the corner reflects the quality of the light guide and readout circuit design. Therefore, it is feasible to choose the data from four corners of the flood histogram to evaluate the flood histogram quality. A flood histogram quality parameter is calculated by the full width at half maximum (FWHM) of the spot and the spot center’s distance to adjacent crystals in both the x and y directions (as shown in Figure 14) using the following formula:

(5) $$Q_{i}{}_{j}=\frac{P_{i}-P_{j}}{({\bar{w}}_{i}+{\bar{w}}_{j})/2}\;\;,$$

where ${\bar{w}}_{i}$ and ${\bar{w}}_{j}$ are the FWHMs of the x or y projections of the *i*th and *j*th crystal; P${}_{i}$ and P${}_{j}$ are the center positions of the *i*th and *j*th crystals. The *i*th and *j*th crystals must be adjacent crystals on the four corners of the flood histogram. There are 16 different combinations of adjacent crystals in four corners. The mean value of the 16 $Q_{i}{}_{j}$ values is utilized to represent the quality of flood histogram.

(6) $$\bar{Q}=\frac{\sum_{i=1,j=2}^{16}Q_{i}{}_{j}}{16}\;\;.$$

**Figure 15:**
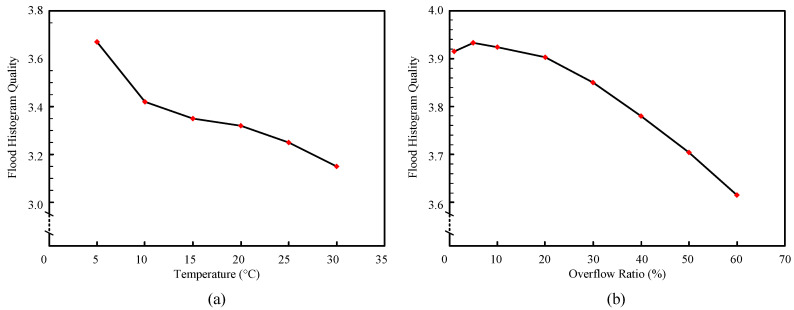
The flood histogram quality parameter values of different temperatures (**a**) and different ADC overflow ratios (**b**).

We calculated the flood histogram quality parameters of different temperature (from 5 ${}^{\circ }C$ to 30 ${}^{\circ }C$, with increments of 5 ${}^{\circ }C$) and different ADC overflow ratios (from 1% to 60%) with using the above evaluation method. Figure 15a shows the parameter values of the flood histogram at different temperatures. The optimal flood histogram was obtained at 5 ${}^{\circ }C$. With the increase of temperature, the quality of flood histogram decreased. Since SiPM is a semiconductor device, it is very sensitive to temperature. On the one hand, for every 1 ${}^{\circ }C$ increase in temperature, the gain of SiPM decreased by 0.8%. On the other hand, the dark current noise of SiPM becomes significantly larger as the temperature rises. That is to say, the signal-to-noise ratio of the output pulse is severely reduced as the temperature increases. The flood histogram was reconstructed by the information extracted from the pulse. Therefore, the spot brightness of the flood histogram becomes darker, and the noise of the flood histogram reconstructed by the pulse becomes larger.

Figure 15b shows the parameter values of the flood histogram with different ADC overflow ratios. With the increase of ADC overflow ratio, the flood histogram quality first increases slightly and then decreases rapidly. The optimal flood histogram quality was obtained when the ADC overflow rate was 5%. When the overflow ratio exceeds a certain value, more and more counts are gathered in the middle of the flood histogram, and the whole flood histogram shows an obvious central convergence phenomenon that can be resolved by the naked eye. A higher ADC overflow ratio will lead to excessive gain, which will exceed the gain adaptive range of ADC, and the top of the pulse will be truncated. As a result, some of the information contained in the pulse is lost, resulting in a decrease in the quality of the reconstructed flood histogram.

**Figure 16:**
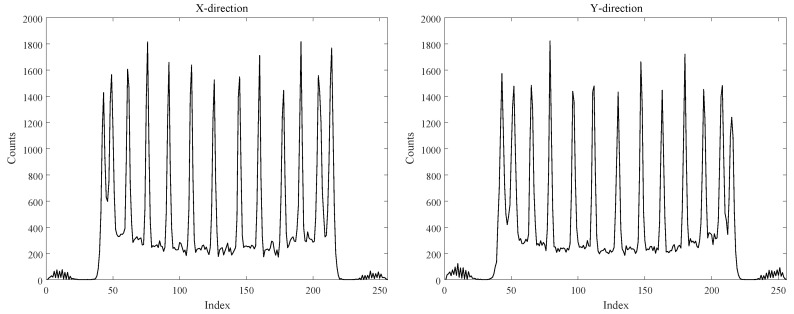
The projection profiles of the x direction and y direction of the flood histogram.

According to the flood histogram evaluation method, the optimal flood histogram quality is obtained with the temperature of 5 ${}^{\circ }C$ and the overflow ratio of 5%. We project the x direction and y direction of the optimal flood histogram, as shown in Figure 16. It is obvious to see the 13 peaks and corresponding peak-valleys in the projection. The optimal spatial resolution of 1.7 mm was obtained by calculating the FWHM of the projection.

### 3.3. Energy Resolution

**Figure 17:**
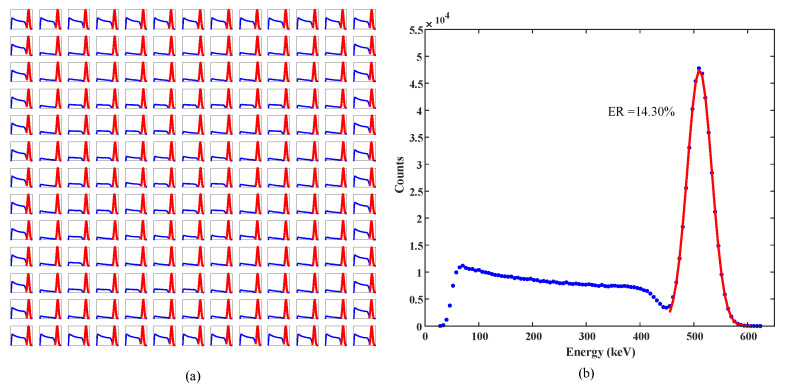
The energy spectra of all the crystal pixels of the 13 × 13 LYSO crystal array (**a**). The average energy spectrum of one LYSO/SiPM block (**b**). An average energy resolution of 14.30% was obtained.

Energy resolution (ER) represents the ability to resolve the photon energy of a PET detector. The scattering events can be filtered out and the deterioration of image quality caused by energy misjudgment can be reduced by setting the energy window. The distribution of pulse height is obtained by statistics of the energy information of the crystal pixels. A Gaussian function is used to fit the photopeak of the pulse height spectrum to obtain the full width at half maximum. The energy resolution is obtained by calculating the ratio of the FWHM of the photopeak to the abscissa of the peak value. Figure 17a shows the energy spectra of all the crystal pixels of the 13 × 13 LYSO crystal array. The central region of the crystal array has the best energy resolution while the energy resolution of the edge is relatively poor. All energy spectra of the crystal array were normalized, and the average energy spectrum was obtained, as shown in Figure 17b. The average energy resolution of the LYSO/SiPM block was 14.30% with a temperature of 5 ${}^{\circ }C$ and overflow ratio of 5%.

### 3.4. Timing Resolution

**Figure 18:**
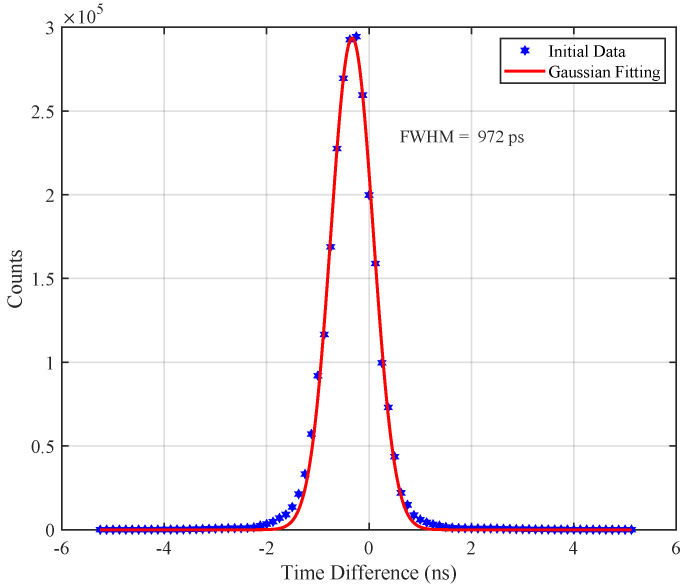
The timing spectrum obtained with a pair of detectors. The coincidence timing resolution of 972 ps obtained by fitting with a Gaussian function.

Time resolution (TR) represents the inaccuracy of the arrival time of a pair of photons after annihilation. An excellent time resolution can greatly improve the image quality. By utilizing the experimental setup shown in Figure 10, we obtained the event data of a pair of detectors working in the coincidence mode. Pick-off the time that the photon arrives at the detector, and the arrival time difference is calculated to obtain the distribution of the time difference of the coincidence detector pair. Ideally, the variance of the measured time difference should be equal to zero, but in fact, it is limited by the time resolution of the detector. The time difference measured is a random variable with broadening, as shown in Figure 18. A Gaussian function is used to fit the photopeak of the time spectrum, and the full width at half maximum of the photopeak is obtained, which means the coincidence time resolution is 972 ps.

## 4. Conclusions and Discussion

This study introduced the composition and design of LIGHTENING${}^{®}$ PET detector in detail. A multiplexing method was proposed to simplify numerous SiPM readout channels and reduce the complexity of the back-end acquisition circuit. A semi-cut light guide with a semi-gap cut was designed and significantly enhanced the resolve ability and performance uniformity of edge crystal pixels. A DTI method with a lesser system hardware cost for digital acquisition of fast scintillation pulse was presented. The gain difference among different SiPM channels was minimized by utilizing the adaptive method based on gain adjustment. A data transmission unit based on UDP protocol was designed to realize data packaging and transmission of the detector module. The performance of the detector was evaluated comprehensively, and we obtained a series of excellent results. Firstly, the flood histograms of using the non-light guide, continuous light guide, and semi-cut light guide were compared. The flood histogram with the semi-cut light guide showed that all the crystal pixels are resolved clearly. Then the flood histograms at different temperatures and different ADC overflow ratios were obtained, respectively. A method of flood histogram quality evaluation was proposed to evaluate the flood histogram quantitatively. The results showed that the best flood histogram quality was obtained when the temperature was 5 ${}^{\circ }C$ and the overflow ratio was 5%. In this case, the SiPM has less dark current noise and the ADC has more dynamic range utilization. Finally, the performance of the detector under the optimal conditions was evaluated and we obtained an optimal spatial resolution of 1.7 mm, average energy resolution of 14.30%, and coincidence timing resolution of 972 ps.

There is no doubt that the LIGHTENING${}^{®}$ PET detector based on LYSO/SiPM has extremely good performance and high research and application value. Compared with the previous work, the work focused on the optimal performance in one aspect. We have obtained better comprehensive performance with a great reduction of system cost. In our next work, we will form a PET system with the detector designed in this paper and evaluate the performance of the whole PET system comprehensively. Furthermore, we can compare other different channel multiplexing methods to further simplify the number of front-end channels. We can study a more accurate time pickoff method to improve the time performance of the detector and develop the PET scanner with TOF information. With the preparation of more new scintillation crystals and the continuous development of SiPM, the performance of the PET detector will be further improved, and the reduction of system cost will be achieved in the near future.

## Figures and Tables

**Table 1:** The specifications and performances of the detectors developed in the previous studies.

	Crystal Array	SiPM Array	ER ${}^{1}$	CTR ${}^{2}$	SR ${}^{3}$
TOF-PET detector [33]	12 × 12	8 × 8	15.6%	514 ps	/
head-mounted micro-dose PET [34]	32 × 32	10 × 10	/	/	2–3 mm
proton therapy monitoring system [35]	6 × 6	6 × 6	24%	/	3 mm
small-scale PET system [36]	24 × 24	8 × 8	13.9%	/	/
proof-of-concept detector [37]	8 × 4	8 × 4	14.6%	495 ps	2.77 mm
DOI-TOF PET module [38]	6 × 6	4 × 4	11.7%	349 ps	3.5 mm
side-by-side phoswich detector [39]	12 × 12	8 × 8	15.6%	/	1.35 mm
Dual-ended readout PET detector [40]	17 × 17	4 × 4	21.0%	1230 ps	0.5 mm

${}^{1}$ Energy resolution (ER). ${}^{2}$ Coincidence time resolution (CTR). ${}^{3}$ Spatial resolution (SR).

**Table 2:** The general specifications of the LIGHTENING${}^{®}$ PET.

	Category	Characteristics
Crystal	Crystal material	LYSO
Block	Crystal size (mm${}^{3}$)	1.92 × 1.92 × 13.00
	Crystal pitch (mm${}^{2}$)	2.02 × 2.02
	Crystal array	13 × 13
BDM	LYSO/SiPM block	1 × 4
	LYSO Dimension (mm${}^{3}$)	26.5 × 106.0 × 13.0
Scanner	Number of BDMs	4
	Number of crystal pixels	2704
	Bore diameter (cm)	15.0
	Transaxial field-of-view (TFOV) (cm)	10.6
	Axial field-of-view (AFOV) (cm)	2.65
